# Kaolin-Assisted Construction of Superhydrophobic Cellulose Aerogels for Recyclable Oil/Water Separation

**DOI:** 10.3390/gels12060529

**Published:** 2026-06-12

**Authors:** Shixue He, Weilong Fei, Ming Shi, Zaijiong Chang, Daning Lang, Ronglan Wu

**Affiliations:** 1 Key Laboratory of Oil & Gas Fine Chemicals, Ministry of Education & Xinjiang Uygur Autonomous Region, School of Chemical Engineering, Xinjiang University, Urumqi 830017, China; 2 School of Mechanical Engineering & Center for Post-Doctoral Studies of Mechanical Engineering, Xinjiang University, Urumqi 830017, China

**Keywords:** cellulose aerogel, kaolin, superhydrophobicity, hydrophobic modification, oil–water separation, recyclability

## Abstract

In recent years, oil spill accidents and oily wastewater discharge have posed severe threats to aquatic ecosystems and human health. Developing green, low-cost, efficient, and recyclable oil–water separation materials is therefore important for environmental remediation. In this work, kaolin/cellulose composite aerogels were fabricated through a low-temperature NaOH/urea dissolution system using N,N′-Methylenebisacrylamide (MBA) as the cross-linking agent, followed by freeze-drying and hydrophobic modification with Methyltrimethoxysilane (MTMS). The structure, morphology, thermal stability, wettability, mechanical behavior, oil adsorption capacity, and reusability of the aerogels were systematically investigated. The composite aerogels exhibited a honeycomb-like interconnected porous structure with low density and high porosity. Kaolin acted as an inorganic reinforcing and roughness-regulating component, which promoted the formation and anchoring of an MTMS-derived siloxane/SiO_2_-like hydrophobic layer on the aerogel surface. The modified aerogels showed superhydrophobicity with a water contact angle above 152° and excellent oleophilicity. The optimized SC3K0.5 aerogel delivered adsorption capacities of 13.5 g/g for pump oil and 12.5 g/g for diesel. After 10 adsorption–desorption cycles, the adsorption capacity remained above 90% of the initial value, indicating good recyclability and mechanical stability. This recyclable kaolin/cellulose aerogel provides a feasible strategy for practical oil–water separation and oily wastewater treatment.

## 1. Introduction

Oil spills and oily wastewater generated from petroleum exploitation, transportation, petrochemical processing, mechanical processing, and domestic discharge have become persistent and difficult-to-remediate environmental problems [1]. Once released into aquatic environments, oil pollutants can rapidly spread over the water surface, block oxygen transfer, inhibit photosynthesis, damage aquatic organisms, and further threaten human health through bioaccumulation and food-chain transfer [2]. More importantly, oily wastewater often contains oils with different densities, viscosities, and chemical compositions, making its efficient collection and separation highly challenging [3]. Therefore, developing green, low-cost, recyclable, and structurally stable materials for oil/water separation is of great significance for environmental remediation and resource recovery.

Various treatment technologies, including gravity separation, flotation, centrifugation, coagulation, membrane separation, and adsorption, have been applied to oily wastewater treatment. Among these approaches, adsorption is regarded as one of the most practical methods because of its simple operation, low energy consumption, high separation efficiency, and suitability for both continuous treatment and emergency oil-spill cleanup [4,5,6]. In particular, adsorbents with special wettability have attracted increasing attention. Inspired by natural superhydrophobic surfaces such as lotus leaves, superhydrophobic/superoleophilic materials can selectively repel water while rapidly adsorbing or transporting oils, thereby providing an effective strategy for selective oil capture and oil/water separation [7,8].

Aerogel-based adsorbents are promising candidates for oil/water separation because of their ultralow density, high porosity, large internal surface area, and interconnected porous networks [9]. These structural features provide abundant storage space, capillary transport channels, and accessible adsorption sites for oils and organic solvents. Compared with synthetic polymer foams [10], carbon aerogels, graphene-based aerogels, and other inorganic porous materials, biomass-derived aerogels show unique advantages in terms of renewability, biodegradability, environmental compatibility, and raw-material availability. Among various biomass resources, cellulose is particularly attractive because it is the most abundant natural polymer on Earth and contains abundant hydroxyl groups that enable dissolution, regeneration, crosslinking, and chemical modification. Accordingly, cellulose-based aerogels have been widely explored as sustainable adsorbents for oil/water separation and organic pollutant removal [11]. Researchers have further confirmed that plant cellulose aerogels, nano-cellulose aerogels and biomass-derived porous materials can provide lightweight structures, interconnected transport channels and adjustable surface wettability, thus achieving efficient oil adsorption and oil–water separation [12,13].

Despite these advantages, pristine cellulose aerogels still face several critical limitations for practical oil/water separation. First, the abundant hydroxyl groups on cellulose chains make the aerogel intrinsically hydrophilic, resulting in poor oil/water selectivity in aqueous environments [14]. Second, the lightweight porous skeleton of cellulose aerogels is prone to shrinkage, collapse, or mechanical damage during drying, compression, and repeated adsorption–desorption cycles [15]. Third, although hydrophobic modification can improve oil selectivity, the introduced low-surface-energy coating may detach from the smooth cellulose skeleton during long-term use, leading to decreased hydrophobicity and reduced recyclability [16]. Therefore, constructing cellulose aerogels with stable superhydrophobicity, robust porous skeletons, and durable surface modification remains a key challenge [17,18].

To address these issues, an effective strategy is to simultaneously regulate the internal skeleton and external surface chemistry of cellulose aerogels [19,20]. On one hand, hierarchical micro/nano-scale roughness is essential for achieving and maintaining superhydrophobicity [21,22]. On the other hand, a stable low-surface-energy layer is required to ensure oil affinity and water repellency [23]. Inorganic fillers provide a feasible way to reinforce the cellulose network and construct rough interfaces. Layered silicate minerals, such as montmorillonite and kaolin, have been used to improve the mechanical properties, dimensional stability, and interfacial structure of polymer-based porous materials. Among them, kaolin is a naturally abundant aluminosilicate mineral with low cost, good chemical stability, plate-like morphology, and abundant oxygen-containing surface groups [24]. Importantly, kaolin with a plate-like and oxygen-rich structure can reinforce the porous cellulose skeletons [25,26], thereby reducing structural collapse and coating detachment during repeated use. These characteristics make kaolin a promising modifier for cellulose aerogels.

The introduction of kaolin into cellulose aerogels is expected to play multiple roles. First, kaolin can serve as an inorganic reinforcing component to improve the structural stability of the cellulose skeleton. Second, the plate-like kaolin particles can introduce micro-scale roughness and increase surface heterogeneity, which is beneficial for constructing superhydrophobic interfaces. Third, the oxygen-containing groups on kaolin may provide additional anchoring sites for subsequent silane modification, thereby improving the stability of the hydrophobic layer. Previous studies have demonstrated that kaolin-based membranes and kaolin-containing composites exhibit potential in oily wastewater treatment because of their low cost, tunable porous structure, and favorable interfacial properties [27,28]. However, the use of kaolin as a roughness-regulating and hydrophobic-layer-anchoring component in cellulose aerogels for recyclable oil/water separation has not been sufficiently explored.

MTMS is a commonly used silane precursor for constructing hydrophobic surfaces [29]. Through hydrolysis and condensation, MTMS can form a siloxane/SiO_2_-like network on hydroxyl-rich substrates, thereby reducing surface energy and enhancing oil affinity. In a kaolin/cellulose composite system, kaolin-induced roughness and MTMS-derived low-surface-energy modification can work synergistically to construct a stable superhydrophobic/superoleophilic surface. Meanwhile, the interconnected honeycomb-like aerogel skeleton can provide efficient capillary channels and oil storage space. Therefore, combining kaolin reinforcement with MTMS hydrophobic modification offers a simple and low-cost route for preparing recyclable cellulose-based oil/water separation materials.

Herein, we report a kaolin-assisted strategy for constructing superhydrophobic cellulose aerogels toward recyclable oil/water separation. By integrating kaolin reinforcement with methyltrimethoxysilane-derived hydrophobic modification, the resulting composite aerogel was designed to simultaneously achieve a robust porous skeleton, hierarchical surface roughness, and a stable low-surface-energy interface. The influence of kaolin loading on the morphology, chemical structure, wettability, mechanical behavior, oil adsorption performance, and recyclability of the aerogels was systematically evaluated. Benefiting from the synergistic effect of the kaolin-induced rough interface and the siloxane/SiO_2_-like hydrophobic layer, the optimized aerogel showed stable superhydrophobicity, favorable compressibility, and efficient cyclic oil adsorption. This work offers a facile, economical, and sustainable route for developing cellulose-based porous adsorbents for oily wastewater treatment and oil-spill remediation.

## 2. Results and Discussion

### 2.1. Gelation Process of Hydrogels

**Figure 1:**
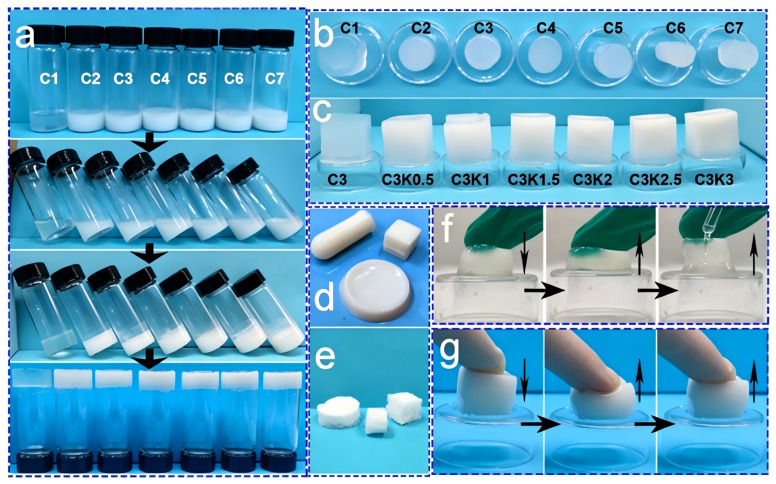
(**a**) Gelation process of hydrogel; (**b**) Cx hydrogel; (**c**) C3Ky hydrogel; (**d**) C3K0.5 hydrogel treated with different molds; (**e**) Aerogel; (**f**) Squeezing diagram of C3 hydrogel; (**g**) Squeezing diagram of C3K0.5 hydrogel.

Taking hydrogel C3Ky as an example, the gelation process of the hydrogel is shown in Figure 1a. After adding the cross-linking agent and stirring uniformly, the mixed solution turned from transparent to white with the addition of kaolin. This color change indicates that kaolin particles were uniformly dispersed in the cellulose solution without obvious agglomeration, and the inorganic–organic composite system was successfully constructed. After cross-linking and gelation, the flowable liquid transformed into a fixed-shape hydrogel, suggesting that the polymerization and cross-linking of MBA proceeded smoothly even with the introduction of kaolin particles. The presence of kaolin did not hinder the gelation behavior but provided additional physical support points through hydrogen bonding and interfacial interaction between kaolin and cellulose chains. The morphology of hydrogels after ion exchange varied with raw material content. Figure 1b,c show optical photographs of Cx hydrogels and C3Ky hydrogels, respectively. It can be seen that when the cellulose content of Cx hydrogel is low, the hydrogel swells to a certain extent after ion exchange, while the addition of kaolin does not affect the shape of the hydrogel before and after ion exchange. This phenomenon reveals that the plate-like kaolin can effectively inhibit the swelling deformation of the hydrogel network during water washing and ion exchange, thereby improving the dimensional stability of the material. Hydrogel gelation is a transition from liquid to semi-solid state, so hydrogels with different shapes can be prepared using different molds (Figure 1d), and aerogels with different shapes can be obtained after freeze-drying (Figure 1e), which can be used for different applications. As shown in Figure 1f,g, the prepared hydrogels displayed good softness and elastic recovery, rapidly restoring their original shape after being compressed to approximately 30% strain. This good elasticity is attributed to the stable three-dimensional network formed by chemical cross-linking of MBA and physical entanglement of cellulose chains [16,30], which lays a mechanical foundation for the subsequent cyclic compression and oil–water separation applications of aerogels.

### 2.2. Morphology and Chemical Composition

**Figure 2:**
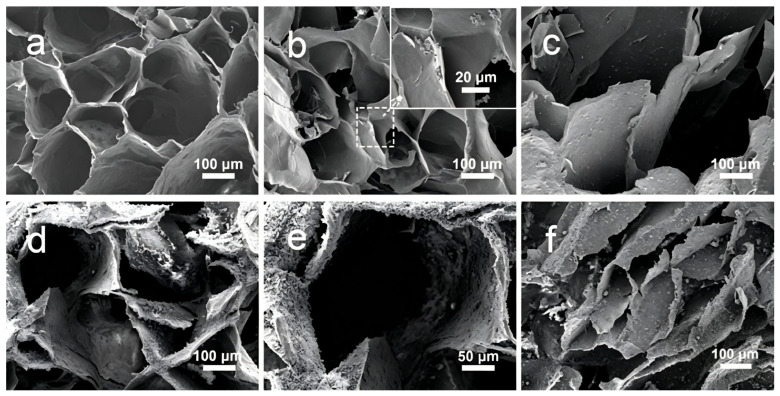
SEM images of aerogels: (**a**) C3; (**b**) SC3; (**c**) C3K0.5; (**d**,**e**) Cross-section and (**f**) Longitudinal section of SC3K0.5.

The microstructure of aerogel adsorbents is the main factor affecting adsorption capacity. As shown in Figure 2a–d, all four aerogels have a micron-scale structure with a honeycomb-like network structure and irregular cross-linking. Pure cellulose (C3) aerogel has a smooth surface and a honeycomb pore structure (Figure 2a); modified cellulose (SC3) aerogel shows little change in surface morphology, with a small amount of SiO_2_ particle loading in some areas (Figure 2b). This indicates that single MTMS modification can only form a thin hydrophobic coating on the surface, but cannot effectively construct hierarchical roughness required for superhydrophobicity. In contrast, C3K0.5 aerogel compounded with kaolin has a rough surface with tiny scale-like protrusions (Figure 2c). Furthermore, SC3K0.5 aerogel modified with kaolin and silane reagent has an even rougher surface (Figure 2d–f). This may be because the rough structure formed by doping kaolin enables the modifier to better combine with the tiny protrusions on the aerogel surface during the reaction, and the generated tiny particles completely cover the aerogel surface, forming a rough surface layer. Moreover, this rough structure is distributed on all sides of the aerogel, including the cross-section (Figure 2d,e) and longitudinal section (Figure 2f), which is beneficial to its hydrophobicity [31]. The interconnected honeycomb pore structure inside the aerogel facilitates liquid storage and transport, providing a structural basis for adsorption and separation.

**Figure 3:**
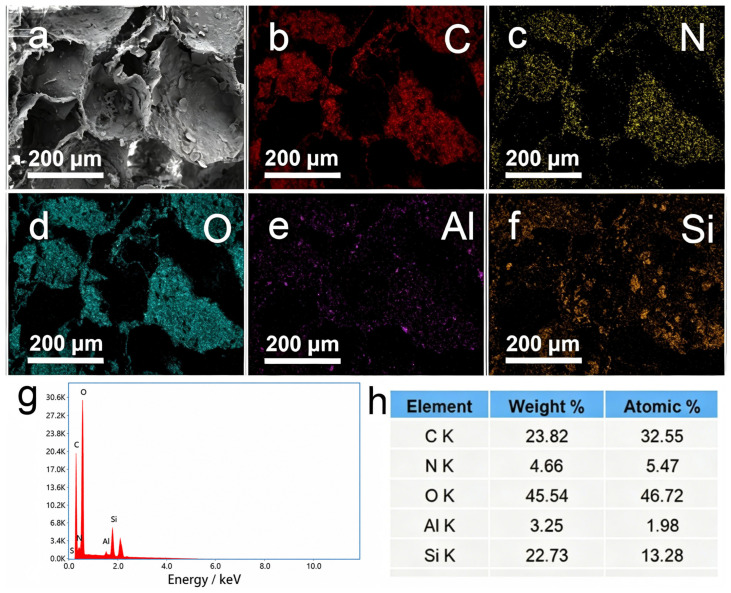
Mapping images of SC3K0.5 aerogel: (**a**) SC3K0.5; (**b**) C; (**c**) N; (**d**) O; (**e**) Al; (**f**) Si; (**g**) EDS spectrum; (**h**) Element content.

Meanwhile, all elements in SC3K0.5 aerogel are uniformly distributed (Figure 3a–f). Oxygen shows the highest relative content, which is associated with the oxygen-containing groups of cellulose and kaolin as well as the hydrolyzed/condensed MTMS-derived siloxane layer (Figure 3g,h). Carbon mainly originates from the cellulose skeleton, nitrogen from the MBA cross-linker, aluminum from kaolin, and silicon from both kaolin and the MTMS-derived siloxane/SiO_2_-like coating. These results suggest that kaolin was successfully incorporated into the cellulose network and that hydrophobic modification was achieved on the composite aerogel surface.

Based on the above structural observations, the role of kaolin can be understood from three aspects. First, kaolin serves as an inorganic reinforcing filler and improves the structural stability of the cellulose aerogel skeleton [32]. Second, its plate-like particles introduce micro-scale roughness, providing more anchoring sites for MTMS hydrolysis and condensation. Third, the roughened interface helps stabilize the siloxane/SiO_2_-like hydrophobic layer during adsorption–desorption cycles, reducing surface coating loss and improving reusability.

**Figure 4:**
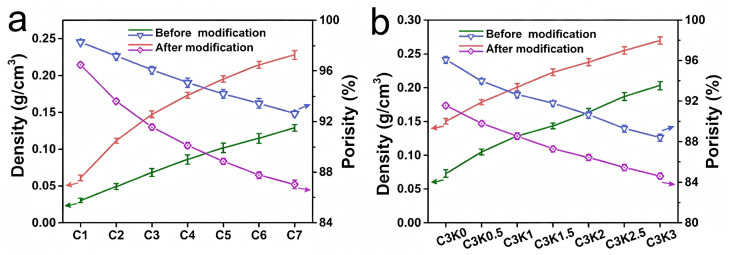
Apparent density and porosity curves of (**a**) Cx and (**b**) C3Ky aerogels before and after modification.

As shown in Figure 4a, the apparent density of Cx aerogels increases with increasing cellulose content, whereas the porosity shows the opposite trend. After MTMS modification, the apparent density of SCx aerogels becomes higher than that of the corresponding unmodified Cx samples, which can be attributed to the formation of a siloxane/SiO_2_-like coating on the aerogel skeleton. Similarly, the apparent density of C3Ky aerogels increases with increasing kaolin content, while the porosity gradually decreases (Figure 4b). These results indicate that cellulose concentration, kaolin loading, and surface modification jointly regulate the lightweight porous structure of the aerogels.

**Figure 5:**
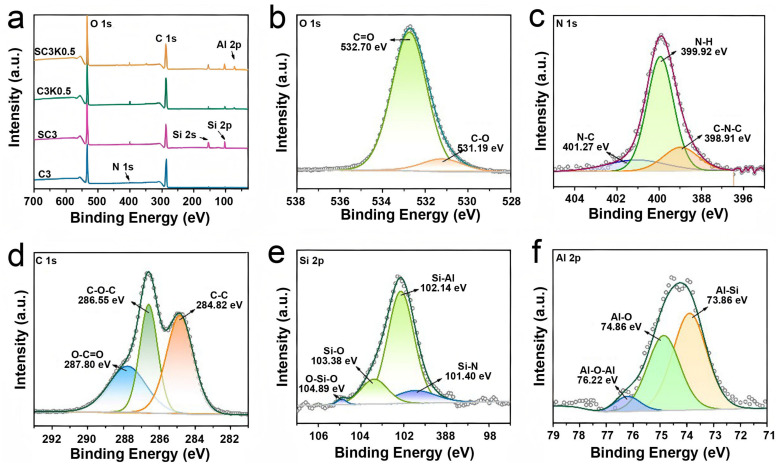
(**a**) XPS full spectra of C3, SC3, C3K0.5 and SC3K0.5 aerogels; High-resolution spectra of SC3K0.5: (**b**) O 1s; (**c**) N 1s; (**d**) C 1s; (**e**) Si 2p; (**f**) Al 2p.

X-ray photoelectron spectroscopy (XPS) was used to further determine the near-surface elemental composition and chemical states of different cellulose aerogels. In the survey spectrum (Figure 5a), the characteristic signals of O 1s, N 1s, C 1s, Si 2s, Si 2p, and Al 2p can be observed. The appearance of Si-related peaks in SC3 confirms the successful introduction of MTMS-derived siloxane species, while the simultaneous presence of Si and Al signals in C3K0.5 and SC3K0.5 verifies kaolin incorporation. Compared with C3K0.5, SC3K0.5 shows stronger Si 2s and Si 2p signals [33], further indicating the formation of a silicon-containing hydrophobic layer after MTMS modification.

The high-resolution O 1s spectrum of SC3K0.5 (Figure 5b) can be assigned to oxygen-containing bonds such as C=O/C-O and Si-O-related species. The N 1s spectrum (Figure 5c) contains N-C, N-H, and C-N-C components, confirming the participation of MBA in the cross-linked cellulose network. The C 1s spectrum (Figure 5d) is mainly fitted with C-C/C-H, C-O/C-O-C, and O-C=O components, which are consistent with the cellulose skeleton and cross-linked structure. The Si 2p spectrum (Figure 5e) is mainly attributed to Si-O-Si (104.89 eV) and Si-O (103.38 eV) bonds, supporting the hydrolysis/condensation of MTMS and the formation of a siloxane/SiO_2_-like surface layer. The Al 2p spectrum (Figure 5f) corresponds to Al-O and Al-Si species from kaolin. Overall, the XPS results confirm the successful construction of a kaolin/cellulose composite aerogel with a silicon-containing hydrophobic surface.

**Figure 6:**
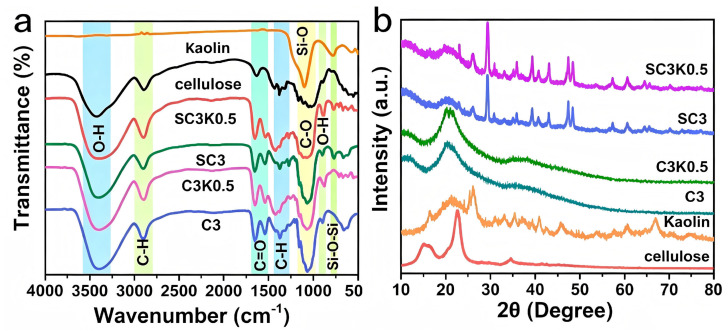
(**a**) FT-IR and (**b**) XRD spectra of aerogels.

FT-IR spectra of cellulose, kaolin, C3, SC3, C3K0.5 and SC3K0.5 aerogels are shown in Figure 6a. The broad absorption band at 3200–3700 cm^−1^ corresponds to O-H stretching vibration, while the peaks at 3000–2850 cm^−1^ are assigned to C-H stretching vibration. The bands in the ranges of 1700–1500 cm^−1^, 1500–1250 cm^−1^, and 1260–1000 cm^−1^ are associated with C=O, C-H bending, and C-O-C/C-O stretching vibrations, respectively, which are typical of cellulose-based networks. After MTMS modification, the enhanced Si-O-related absorption around 1093 cm^−1^ indicates the formation of a siloxane/SiO_2_-like layer on the aerogel skeleton [34]. The characteristic kaolin-related Si-O and Al-O vibrations further support the successful incorporation of kaolin into the composite aerogels. Figure 6b shows XRD patterns of cellulose, kaolin, C3, SC3, C3K0.5 and SC3K0.5 aerogels. The characteristic diffraction peaks of cellulose appear at 2θ = 16.63° and 23.15°, corresponding to the typical cellulose crystal planes [35]. After cross-linking and kaolin incorporation, the cellulose-related peaks become slightly broadened and shifted, indicating that the dissolution-regeneration process and composite formation influence the crystalline domains of cellulose. For SC3 and SC3K0.5, the enhanced silicon-related signals can be associated with the MTMS-derived siloxane/SiO_2_-like layer and the inorganic kaolin component. Considering the mild modification temperature, the silicon-containing phase is more reasonably described as a surface siloxane/SiO_2_-like coating rather than abundant highly crystalline SiO_2_. Therefore, the XRD results, together with FT-IR and XPS analyses, confirm the successful construction of kaolin-containing and hydrophobically modified cellulose aerogels.

**Figure 7:**
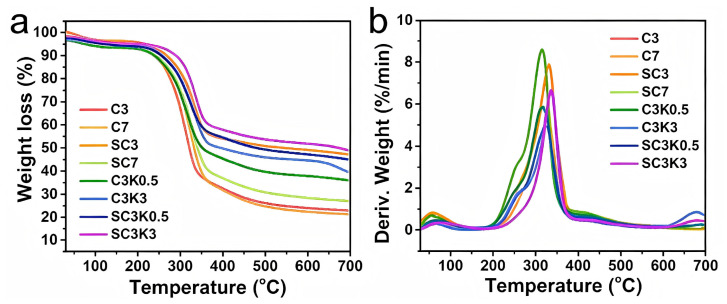
(**a**) TG curves of different aerogels; (**b**) DTG curves of different aerogels.

Thermogravimetric analysis of aerogels is shown in Figure 7a,b. The thermal degradation behavior of all samples can be divided into three main stages, with key parameters summarized in Table 1 for clearer comparison. In the range of 25–100 °C, all aerogels exhibit slight mass loss, which is mainly attributed to the evaporation of physically adsorbed water, indicating that the aerogel structure remains stable during drying at 70 °C. A minor mass loss at 100–200 °C may be related to the removal of bound water. The rapid mass loss between 200 and 400 °C and the DTG peak near 350 °C mainly originate from the decomposition and dehydration of the cellulose skeleton [36]. For pure cellulose aerogels (C3, C7), the initial decomposition temperature is around 220–230 °C, and the temperature of maximum mass loss rate is approximately 300–310 °C. After kaolin incorporation (C3K0.5, C3K3) and MTMS modification (SC3, SC3K0.5, SC3K3), both the initial decomposition temperature and the temperature of maximum mass loss rate shift to higher temperatures: the initial decomposition temperature increases to approximately 235–250 °C, and the temperature of maximum mass loss rate shifts to approximately 310–340 °C, indicating improved thermal stability. After 400 °C, the mass loss rate slows significantly, the residual mass tends to stabilize. The modified and kaolin-containing aerogels show higher residual mass than pure cellulose aerogels. This enhancement is attributed to two factors: (1) the inorganic kaolin filler acts as a thermal barrier, limiting heat transfer and inhibiting the decomposition of the cellulose matrix [37]; (2) the MTMS-derived siloxane surface layer forms a protective char layer during pyrolysis, which suppresses further mass loss. Notably, the residual mass increases with increasing kaolin content, confirming that kaolin plays a dominant role in improving the thermal stability of the composite aerogels. Thus, kaolin incorporation and MTMS modification contribute to the thermal stability of the composite aerogels.

### 2.3. Surface Wettability Analysis of Aerogels

**Figure 8:**
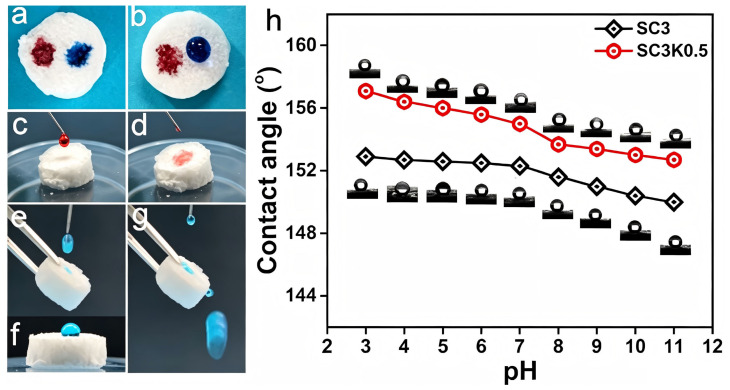
Wettability behavior of aerogels: (**a**,**b**) water and chloroform droplets on C3K0.5 before hydrophobic modification; (**c**–**g**) hydrophobic–oleophilic images of SC3K0.5; (**h**) contact angle curves and optical photos of SC3 and SC3K0.5 under water droplets with different pH values.

To study the wettability of C3Ky aerogels before and after modification to water and organic solvents, optical images of droplet changes on C3K0.5 and SC3K0.5 aerogel surfaces were recorded (Figure 8a,b). Water droplets are rapidly absorbed into C3K0.5 aerogel upon contact, while chloroform droplets are also quickly absorbed, indicating hydrophilic and oleophilic properties of C3K0.5. After hydrophobic modification, water droplets are repelled and remain stable on SC3K0.5 surface, showing hydrophobicity; chloroform droplets are instantly absorbed, confirming hydrophobic–oleophilic properties of SC3K0.5 and successful modification (Figure 8c–g). To investigate the effect of different aqueous environments on the hydrophobic stability of SC3K0.5 aerogel, contact angles under solutions with different pH values were tested (Figure 8h). SC3K0.5 exhibits higher contact angles than SC3 under all tested pH conditions. The contact angle decreases gradually as the solution becomes more alkaline, which may be attributed to partial hydrolysis or erosion of the silicon-containing hydrophobic layer under alkaline conditions. Nevertheless, SC3K0.5 still maintains a contact angle above 152° under alkaline conditions, demonstrating good pH-tolerant hydrophobicity. This excellent stability benefits from the anchoring effect of kaolin, which enhances the adhesion between the hydrophobic layer and the aerogel skeleton, thereby slowing down the damage of the coating in harsh environments.

### 2.4. Compressive Properties of Aerogels

**Figure 9:**
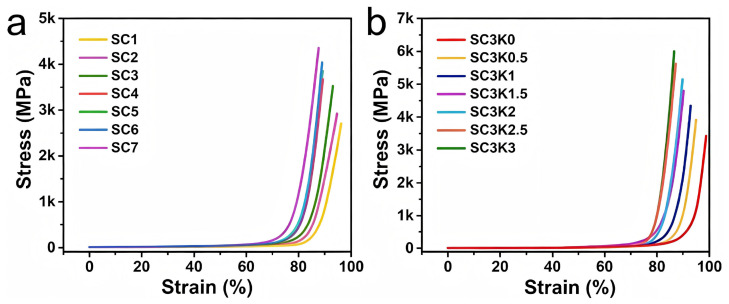
(**a**) Compression strain curves of modified SCx aerogels; (**b**) Compression strain curves of modified SC3Ky aerogels.

Excellent elasticity and compressibility are critical for oil recovery and reuse in practical applications. Figure 9 shows the compressive strain curves of SCx and SC3Ky aerogels. As shown in Figure 9a, the compressive strength of SCx aerogels increases with compressive strain, but the variation with cellulose content is not fully regular, which may be related to differences in pore uniformity and partial loss of the surface coating during compression [38]. In contrast, kaolin doping enhances the compressive response of SC3Ky aerogels (Figure 9b). The micro-roughened kaolin-containing skeleton provides more stable anchoring sites for the MTMS-derived surface layer, thereby reducing coating detachment during repeated compression. This improved compressibility is beneficial for oil recovery by mechanical squeezing and for repeated adsorption–desorption cycles. The enhanced mechanical performance ensures that the aerogel can maintain structural integrity after multiple extrusion-regeneration processes, which is essential for long-term cyclic use.

### 2.5. Oil–Water Separation Performance Test of Aerogels

**Figure 10:**
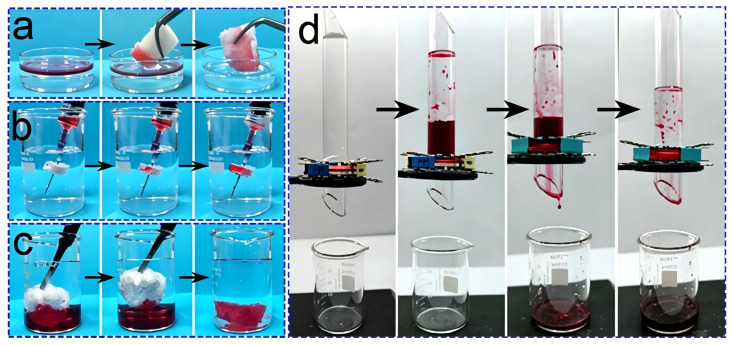
(**a**) Adsorption of floating oil on water by SC3K0.5; (**b**) Simulated adsorption and collection of underwater oil leakage by SC3K0.5; (**c**) Adsorption of underwater heavy oil by SC3K0.5; (**d**) Separation of heavy oil and water using SC3K0.5 as separation membrane.

Figure 10 illustrates the adsorption and oil/water separation behaviors of SC3K0.5 aerogels in different application scenarios. As shown in Figure 10a, the cubic SC3K0.5 aerogel rapidly removed n-hexane stained with Sudan III from the water surface and remained floating after adsorption. This behavior can be attributed to its ultralow density, high porosity, and superhydrophobic/superoleophilic surface, which enable efficient capture of floating oil. As shown in Figure 10b, the cylindrical SC3K0.5 aerogel was able to directionally collect leaked organic liquid underwater under the assistance of an external force. With the guidance of a syringe, the leaked oil was rapidly absorbed by the aerogel without obvious dispersion, indicating its potential for the controlled collection of underwater oil leakage. This result suggests that the aerogel may be useful for emergency treatment of underwater oil spills. Figure 10c further shows that the cylindrical SC3K0.5 aerogel could rapidly adsorb Sudan III-dyed chloroform from underwater. Once the aerogel came into contact with chloroform, the liquid was quickly drawn into the porous framework and removed, demonstrating that the material is applicable not only to floating light oils but also to high-density organic liquids sinking below the water surface. In addition, Figure 10d shows that SC3K0.5 could be assembled into a separation device for gravity-driven oil/water separation. Owing to its selective wettability and interconnected porous structure, chloroform preferentially passed through the aerogel under gravity, while water was retained above the separation layer. Moreover, the SC3K0.5 aerogel exhibited a separation efficiency of 99.8% and a flux of 885.7 L m^−2^ h^−1^ for chloroform/water separation. This result demonstrates that SC3K0.5 can serve not only as an adsorbent for oil collection but also as a separation medium for oil/water mixtures. Overall, the above experiments demonstrate that SC3K0.5 possesses shape tunability while maintaining effective oil/water separation functionality. The aerogel can be used as an adsorbent for collecting floating oil and underwater organic liquids, and it can also function as a separation medium after structural shaping. These features highlight the versatility of the material and broaden its potential applications in oil spill cleanup and oily wastewater treatment.

### 2.6. Oil Absorption Performance Test of Aerogels

**Figure 11:**
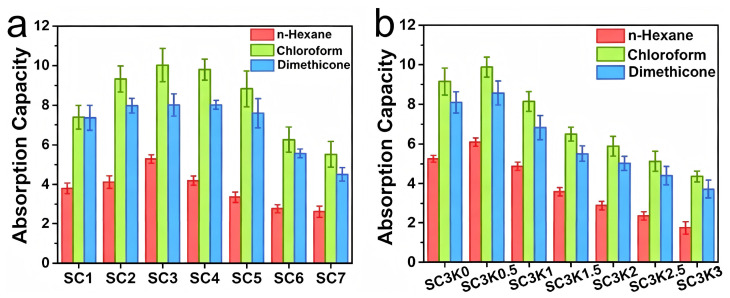
(**a**) Adsorption capacity histogram of modified SCx aerogels for three simulated oils; (**b**) Adsorption capacity histogram of modified SC3Ky aerogels for three simulated oils.

Oil adsorption tests were conducted on SCx and SC3Ky aerogels using three representative organic liquids (Figure 11). The adsorption capacity is affected by the density, viscosity, volatility, and interaction between the liquid and the porous aerogel skeleton. Figure 11a shows that the adsorption capacity of SCx aerogels first increases and then decreases with cellulose content, indicating the existence of an optimal pore structure. Excessively large pores are unfavorable for oil retention, whereas overly dense pores reduce oil storage space and transport efficiency [39,40]. SC3 exhibits adsorption capacities of 10.3 g/g for chloroform, 8.4 g/g for dimethicone, and 5.4 g/g for n-hexane. Figure 11b shows that kaolin loading strongly influences adsorption performance. SC3K0.5 shows better adsorption than SC3, with adsorption capacities of 10.0 ± 0.5 g/g for chloroform, 9.0 ± 0.5 g/g for dimethicone, and 6.5 ± 0.5 g/g for n-hexane. This improvement is attributed to kaolin-induced surface roughness, enhanced coating stability, and optimized porous structure.

**Figure 12:**
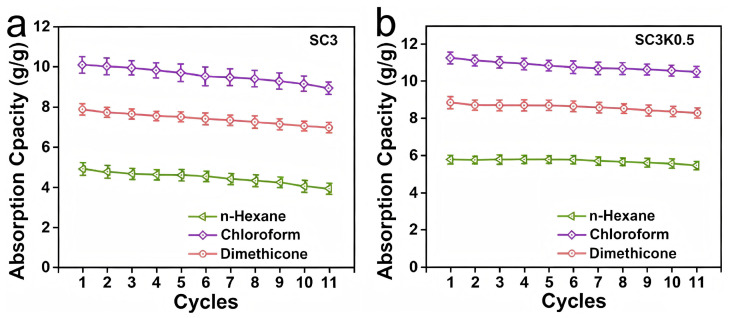
(**a**) Recycling adsorption capacity curves of modified SC3 aerogel for three simulated oils; (**b**) Recycling adsorption capacity curves of modified SC3K0.5 aerogel for three simulated oils.

Recycling adsorption tests were performed on SC3 and SC3K0.5 aerogels using three organic reagents. Figure 12a shows that the adsorption capacity of SC3 gradually decreases with increasing cycle number, retaining approximately 85% for dimethicone and approximately 80% for chloroform and n-hexane after 10 cycles. SC3K0.5 shows a similar decreasing trend (Figure 12b), but it retains approximately 90% of its initial capacity after 10 cycles, indicating better reusability. This enhanced cyclic stability is attributed to the kaolin-induced roughened skeleton, which helps anchor the MTMS-derived hydrophobic layer and reduce surface coating loss during repeated adsorption and squeezing.

**Figure 13:**
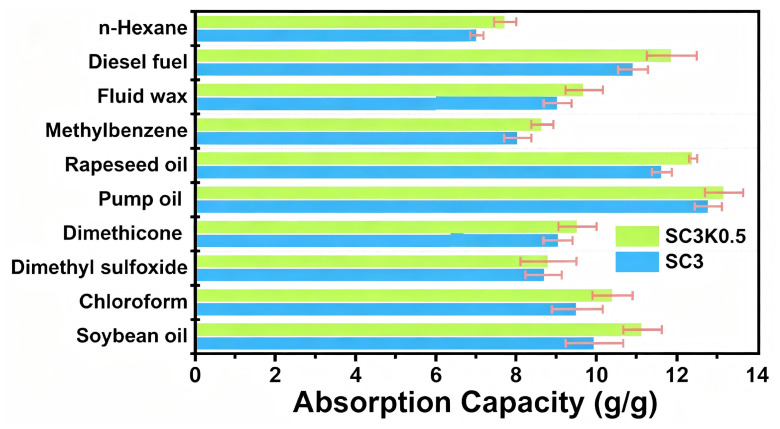
Adsorption capacity histogram of modified SC3 and SC3K0.5 aerogels for ten simulated oils.

Adsorption tests on ten organic reagents (Figure 13) show that both SC3 and SC3K0.5 can adsorb various oils and organic solvents, with adsorption capacity depending on density, viscosity, volatility, molecular size, and pore accessibility [41]. SC3K0.5 outperforms SC3 for all tested liquids, confirming the positive role of kaolin incorporation. Pump oil shows the highest adsorption capacity because of its relatively high molecular weight, high viscosity, and non-volatility, whereas n-hexane shows the lowest measured adsorption capacity due to its high volatility, small molecular size, low density, and low viscosity. The measured adsorption values of volatile liquids may be underestimated because of evaporation during weighing. For high-viscosity oils, the adsorption capacities of SC3 and SC3K0.5 become closer, indicating that liquid viscosity and diffusion resistance also influence the adsorption process. Physical properties of the ten oils are listed in Table 2.

In addition, compared with numerous carbon-based materials, fluorinated materials, and noble metal-based inorganic porous adsorbents, the SC3K0.5 aerogel prepared in this study has a significant cost advantage. This aerogel, made from abundantly available cellulose and low-cost kaolin, performs comparably to previously reported cellulose-based aerogels in terms of water contact angle, oil adsorption capacity, and cyclic reuse performance (Table 3), and is expected to provide a low-cost, sustainable preparation route for recyclable oil–water separation aerogels.

## 3. Conclusions

In summary, this work developed a kaolin-assisted interfacial roughening and hydrophobic-layer anchoring strategy for constructing recyclable superhydrophobic cellulose aerogels. Kaolin was introduced as a multifunctional mineral component to reinforce the cellulose skeleton, regulate surface roughness, and stabilize the MTMS-derived siloxane/SiO_2_-like hydrophobic layer. The resulting SC3K0.5 aerogel exhibited a honeycomb-like interconnected porous structure, stable superhydrophobicity, good oleophilicity, and favorable compressibility. Benefiting from the synergistic effect of kaolin-induced roughness, low-surface-energy modification, and the porous cellulose framework, SC3K0.5 maintained water contact angles above 155° under acidic/neutral conditions and above 150° under alkaline conditions. It achieved adsorption capacities of 13.5 g/g for pump oil and 12.5 g/g for diesel, while retaining approximately 90% of its initial adsorption capacity after 10 adsorption–desorption cycles. The aerogel also showed capability for floating oil adsorption, underwater heavy oil capture, and gravity-driven oil/water separation. This study provides a simple, low-cost, and sustainable route for designing durable biomass-based superhydrophobic adsorbents for oil-spill cleanup and oily wastewater treatment. Future work will focus on evaluating its long-term durability, anti-fouling behavior, and continuous separation performance in complex oily wastewater containing salts, surfactants, and emulsified oil droplets.

## 4. Materials and Methods

### 4.1. Materials

Cellulose, sodium hydroxide (NaOH), urea, and kaolin (Particle size: 2–5 μm) were purchased from Tianjin Zhiyuan Chemical Reagent Co., Ltd. (Tianjin, China); N,N′-methylene bisacrylamide (MBA), and methyltrimethoxysilane (MTMS) were purchased from Tianjin Guangfu Technology Development Co., Ltd. (Tianjin, China); Dimethicone, dimethyl sulfoxide, and liquid paraffin were purchased from Shanghai Aladdin Chemical Co., Ltd. (Shanghai, China); Chloroform was purchased from Chengdu Kelong Chemical Co., Ltd. (Chengdu, China); Toluene, n-hexane, and cyclohexane were purchased from Tianjin Baishi Chemical Co., Ltd. (Tianjin, China); Rapeseed oil, soybean oil were purchased from Guangzhou Jinwang Chemical Co., Ltd. (Guangzhou, China); Diesel fuel was purchased from Sinopec Corp. (Beijing, China); Pump oil was purchased from Xinjiang Fuke Oil Co., Ltd. (Xinjiang, China); Castor oil was purchased from Tianjin Oubokai Chemical Co., Ltd. (Tianjin, China).

### 4.2. Preparation of Cellulose Aerogels (Cx)

A 100 g NaOH/urea aqueous solution with a NaOH/urea/H_2_O mass ratio of 7:13:80 was prepared and pre-cooled before use. A certain mass of cellulose (4.0, 4.5, 5.0, 5.5, 6.0, 6.5 and 7.0 g) was dispersed in the solution and frozen at low temperature (−20 °C) for 12 h to obtain a homogeneous cellulose solution. Then, 25 g of the cellulose solution was mixed with 0.4 g MBA and stirred at room temperature for 3 h. The mixture was poured into a mold and kept at room temperature for 3 h for gelation and demolding, yielding cellulose hydrogels (denoted as C1–C7). The hydrogels were soaked in deionized water until the pH reached 7 to remove residual NaOH and urea. After freezing at −20 °C for 12 h and freeze-drying at −50 °C for 48 h, a series of cellulose aerogels (Cx) were obtained.

### 4.3. Preparation of Kaolin/Cellulose Composite Aerogels (C3Ky)

Kaolin/cellulose composite aerogels were prepared following the same procedure as pure cellulose aerogels with additional kaolin incorporation. Taking the C3 cellulose solution (25 g) as a matrix, 0.5, 1.0, 1.5, 2.0, 2.5, and 3.0 g of kaolin were added separately and stirred thoroughly for 1 h. Then, 0.4 g MBA was added, and the mixture was stirred, molded, and gelled under the same conditions. The subsequent washing, freezing, and freeze-drying steps were identical to those of Cx aerogels, producing kaolin/cellulose composite aerogels denoted as C3Ky, where y represents the mass of kaolin.

### 4.4. Preparation of Hydrophobic Modified Aerogels (SCx and SC3Ky)

**Figure 14:**
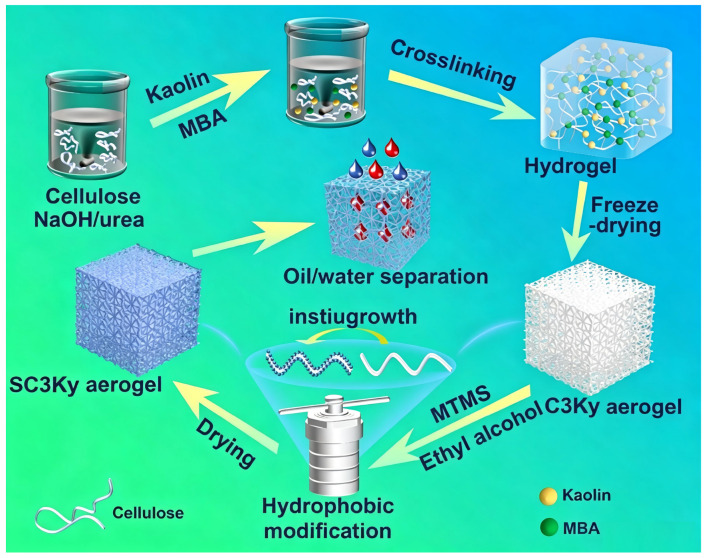
Schematic diagram of the preparation of kaolin/cellulose (SC3Ky) aerogel.

First, 5 mL deionized water and 5 mL ethanol were mixed uniformly in a Teflon-lined autoclave, followed by adding 0.5 mL MTMS and stirring for 10 min to prepare the modification solution. The Cx or C3Ky aerogels were immersed in the solution and reacted at 80 °C for 2 h. After cooling to room temperature, the samples were washed with water and ethanol three times each and dried at 70 °C to obtain hydrophobic modified cellulose aerogels (SCx) and modified kaolin/cellulose composite aerogels (SC3Ky). The preparation process is illustrated in Figure 14.

### 4.5. Characterization and Performance Tests

The surface morphology of the samples was observed by scanning electron microscope (SEM, MERLIN Compact, Zeiss, Oberkochen, Germany) with an electron acceleration voltage of 20 kV. Functional groups of the materials were tested by Fourier transform infrared spectrometer (FT-IR, VERTEX 70, Bruker, Mannheim, Germany) using KBr pellets, with a scanning range of 400–4000 cm^−1^. Thermal stability of the samples was tested by thermogravimetric analysis (TG, SDTQ600, TA Instruments, New Castle, DE, USA) under nitrogen atmosphere, with a heating rate of 10 °C/min from 25 to 800 °C. Crystal structure of the samples was analyzed by X-ray powder diffraction (XRD, D8 Focus, Bruker, Mannheim, Germany) using Cu Kα radiation (wavelength = 0.15418 nm), with a scanning range of 10–80°. Surface elemental composition and chemical states were analyzed by X-ray photoelectron spectroscopy (XPS, ESCALAB 250Xi, Thermo Fisher Scientific, Waltham, MA, USA) using Al Kα radiation. Water and oil contact angles on the sample surface were measured by a contact angle tester (JC2000D4, Zhongchen, Shanghai, China). Compressive strength of the aerogel was measured by a static mechanical tester (H5K-T, Tinius Olsen, Horsham, PA, USA) at a compression speed of 50 mm/min.

### 4.6. Apparent Density and Porosity Calculation

The apparent density (*ρ*) and porosity (*P*) of the aerogels were calculated according to Equations (1) and (2) [44], respectively:

(1) $$\rho =\frac{4m}{\pi d^{2}h}$$

(2) $$P(\%)=(1-\frac{\rho }{\rho_{s}})\times 100$$

where *ρ* is the aerogel apparent density (g/cm^3^), *m* is the aerogel mass (g), d is the aerogel diameter (cm), h is the aerogel height (cm), *P* is the aerogel porosity (%), and *ρ*_s_ is the density of cellulose (g/cm^3^). Unless otherwise specified, all measurements were conducted using three independent samples.

### 4.7. Oil Adsorption and Oil–Water Separation Performance

Aerogels were immersed in various oils/organic solvents until saturation, then removed and drained for 5 s. The adsorption capacity of the aerogel for oils or organic solvents was calculated using Equation (3) [45].

(3) $$Q=\frac{m-m_{0}}{m_{0}}$$

where *m* is the mass of the aerogel after adsorption (g), m_0_ is the initial mass of the aerogel (g), and *Q* is the saturated adsorption capacity (g/g). Unless otherwise specified, all measurements were conducted using three independent samples.

After mixing 10 mL of water and 10 mL of chloroform evenly, pour it into the separation device containing the aerogel, and test the aerogel’s oil–water separation efficiency and separation flux through Equation (4) and Equation (5) [46], respectively.

(4) $$\mathrm{Separation}\;\mathrm{efficiency}=\frac{C_{0}-C_{1}}{C_{0}}$$

(5) $$\mathrm{Flux}=\frac{V}{At}$$

where *C*_0_ and *C*_1_ are the water concentrations in the feed and filtrate, respectively. *V* is the volume of the filtrate, *A* is the effective area of the sponge, and *t* is the separation time. Unless otherwise specified, all measurements were conducted using three independent samples.

## Figures and Tables

**Table 1:** Thermal stability parameters of the prepared aerogels.

Aerogel	Initial Decomposition Temperature (°C)	Temperature of Maximum Mass Loss Rate (°C)	Residual Mass (%)
C3	222	302	23
C7	225	305	22
SC3	235	312	48
SC7	238	315	27
C3K0.5	230	320	36
C3K3	245	330	39
SC3K0.5	240	328	45
SC3K3	250	336	50

**Table 2:** Physical parameters of ten oils and organic solvents.

Reagent Name	Density (g/cm^3^)	Viscosity (mPa·s)	Volatility
Pump oil	0.88	61.2	Non-volatile
Rapeseed oil	0.92	13.5	Non-volatile
Soybean oil	0.915	8.5	Non-volatile
Liquid paraffin	0.9	110	Slightly volatile
Dimethicone	1	102	Non-volatile
Diesel fuel	0.84	0.56	Non-volatile
Chloroform	1.48	0.563	Highly volatile
Toluene	0.866	0.587	Highly volatile
n-Hexane	0.66	0.307	Highly volatile
Dimethyl sulfoxide	1.1	2.2	Slightly volatile

**Table 3:** Comparison of oil–water separation performance between this work and reported works.

Aerogels	WCA (°)	Max Oil Adsorption (g/g)	Cycles Retention	Refs.
Cellulose nanfibers/sodium alginate aerogels	144.5	41–89	20 cycles 86.4%	[5]
Cellulose/MBA/tannin composite materials	121.5	3–9	10 cycles > 75%	[16]
Microfibrils/regenerated cellulose carbon aerogels	151.5	65–133	25 cycles > 91%	[42]
Cellulose/sodium alginate aerogel	112.5	6.87–8.98	-	[43]
SC3K0.5 aerogel	152	6~14	10 cycles > 90%	This work

## Data Availability

The original contributions presented in this study are included in the article. Further inquiries can be directed to the corresponding author.
